# Role of Sodium-Glucose Transport Protein 2 (SGLT2) Inhibitors in Preventing Cardiac Surgery-Associated Acute Kidney Injury: A Scoping Review

**DOI:** 10.7759/cureus.86630

**Published:** 2025-06-23

**Authors:** Hasan Al Chalabi, Abdousamad Said Omar, Amy Gordon, Bibeka Rai

**Affiliations:** 1 Emergency Medicine, Aneurin Bevan University Health Board, Cwmbran, GBR; 2 General Medicine, Aneurin Bevan University Health Board, Newport, GBR; 3 Medicine and Surgery, Aneurin Bevan University Health Board, Newport, GBR; 4 Geriatric Medicine, Aneurin Bevan University Health Board, Abergavenny, GBR

**Keywords:** acute kidney injury, cardiac surgery, dapagliflozin, empagliflozin, sglt2 inhibitors

## Abstract

Cardiac surgery-associated acute kidney injury (CSA-AKI) is a frequent and serious complication associated with increased morbidity, mortality, and healthcare costs. Sodium-glucose co-transporter 2 inhibitors (SGLT2i), including dapagliflozin and empagliflozin, have shown renoprotective benefits in chronic settings, but their perioperative role in CSA-AKI prevention is not well defined. This study was to map the current literature on the use of SGLT2 inhibitors such as empagliflozin and dapagliflozin for the prevention of CSA-AKI, identify evidence gaps, and inform future research directions. A scoping review was conducted using PubMed, Ovid (MEDLINE/Embase), Cochrane Library, and trial registries from January 2010 to May 2025. Eligible studies included adult cardiac surgery patients receiving SGLT2i therapy in the perioperative period. Data were extracted on study design, SGLT2i agent, timing, AKI definitions, outcomes, and safety reporting. Seven studies met the inclusion criteria, and one early-terminated trial met the inclusion criteria. The terminated study halted recruitment after achieving its predefined outcome and transitioned into a larger multicentre trial. Empagliflozin was the most frequently studied agent, with dapagliflozin included in a smaller subset. Findings suggest a lower incidence of CSA-AKI in patients receiving SGLT2i compared to controls; however, heterogeneity in study design, small sample sizes, and inconsistent safety reporting limit the strength of conclusions. Three ongoing randomized controlled trials were also identified, reflecting growing interest in this therapeutic strategy. Early data suggest that SGLT2 inhibitors may offer renoprotective effects in the cardiac surgical population. Evidence specific to dapagliflozin remains limited. Larger, high-quality RCTs are needed to determine efficacy, safety, and the optimal timing and perioperative use of SGLT2i to prevent CSA-AKI.

## Introduction and background

Cardiac-surgery-associated acute kidney injury (CSA-AKI) affects 20-30% of patients undergoing coronary artery bypass grafting (CABG) or valve surgery and significantly increases morbidity, mortality, and long-term renal impairment [1]. According to the Kidney Disease Improving Global Outcomes (KDIGO) criteria, AKI is defined by a decline in urine output (UO), an increase in serum creatinine (SC), or both [2]. CSA-AKI is associated with increased morbidity and mortality, increased healthcare costs, and prolonged hospital stays [3]. CABG is still the most performed cardiothoracic surgery worldwide [4], and up to half of the surgeries are performed on individuals with type 2 diabetes mellitus (T2DM) [5].

Current strategies to prevent CSA-AKI include risk stratification to identify high-risk patients; optimisation of comorbid conditions, particularly diabetes, anemia, and volume status; medication management such as discontinuation or adjustment of nephrotoxic medications (e.g., nonsteroidal anti-inflammatory drugs or NSAIDs, renin-angiotensin-aldosterone system or RAAS inhibitors) and preoperative hydration [6].

Sodium-glucose cotransporter-2 inhibitors (SGLT2i) have demonstrated robust cardiovascular and renal benefits in outpatient populations with type 2 diabetes, heart failure, and chronic kidney disease [7]. These effects are largely attributed to well-characterized mechanisms, including enhanced tubuloglomerular feedback, reduction in oxidative stress, and improved renal oxygenation, which have been validated in both clinical and experimental studies [8,9]. However, in the perioperative setting, particularly in cardiac surgery, these mechanisms remain theoretical. Preclinical data suggest that SGLT2i activate nutrient-deprivation signalling pathways (such as AMPK and sirtuins), leading to increased autophagy and reduced inflammatory and oxidative injury, which may be renoprotective [8].

This scoping review primarily aims to map the current landscape of completed and ongoing studies investigating the use of SGLT2i for the prevention of CSA-AKI. Secondarily, it seeks to summarize reported outcomes, assess safety signals, and identify key gaps to guide future research.

## Review

Methods

Protocol

This review followed the Joanna Briggs Institute methodology for scoping reviews and the Preferred Reporting Items for Systematic reviews and Meta-Analyses extension for Scoping Reviews (PRISMA-ScR) reporting guideline [10,11]. This review does not include any patient-collected/investigated directly by the authors, so formal ethical approval was not necessary.

Eligibility Criteria

We included studies involving adult (age ≥18 years) patients undergoing cardiac surgery (CABG, valve surgery, or transcatheter aortic valve implantation {TAVI}) who were exposed to any licensed SGLT2 inhibitor (dapagliflozin, empagliflozin, canagliflozin, ertugliflozin) in the perioperative period. Eligible designs included randomized controlled trials, prospective or retrospective cohort studies, and pilot trials. Outcomes of interest included AKI incidence or severity using standard definitions (KDIGO; Risk, Injury, Failure, Loss of kidney function, and End-stage kidney disease {RIFLE}; Acute Kidney Injury Network {AKIN}), and secondary outcomes such as Major Adverse Cardiovascular Events (MACE), Major Adverse Kidney Events (MAKE), all-cause mortality, atrial fibrillation, and safety [2,12,13].

We excluded animal or in vitro studies, non-cardiac surgery, narrative reviews, and editorials.

Information Sources and Search Strategy

We systematically searched PubMed, Ovid (MEDLINE/Embase), and the Cochrane Library from January 2010 to May 24, 2025. Additional sources included ClinicalTrials.gov. A combination of specific keywords and medical subject headings (MeSH), including: dapagliflozin OR SGLT2 inhibitor OR empagliflozin OR canagliflozin OR ertugliflozin AND acute kidney injury OR AKI AND cardiac surgery OR cardiopulmonary bypass OR CABG OR valve surgery. No language restrictions were applied.

The search strategies are detailed in the appendices (see Appendix 1-3).

Selection Process

**Figure 1:**
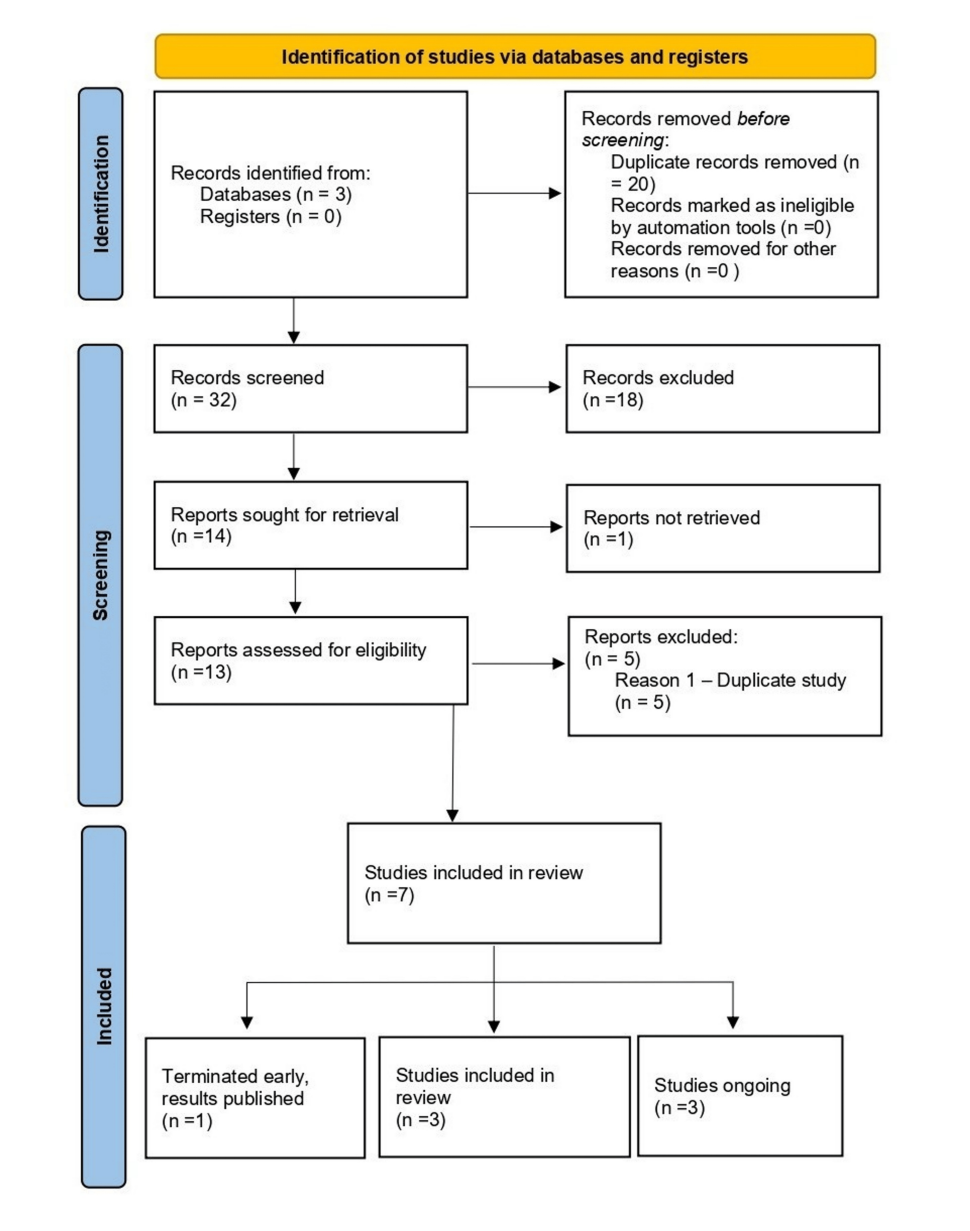
PRISMA flow chart PRISMA: Preferred Reporting Items for Systematic Reviews and Meta-Analyses

We employed Rayyan (Rayyan Systems Inc., Doha, Qatar) to screen and deduplicate all the studies that were identified during our online search. Two reviewers independently screened titles and abstracts. Full texts of potentially eligible studies were reviewed, and inclusion disagreements were resolved through a third author. The PRISMA 2020 flow diagram is presented in Figure 1.

Data Charting

A standardized data extraction form was developed and piloted. Key variables extracted included study year, design, population, data collection period, type and timing of SGLT2 inhibitors exposure, AKI definitions, incidence and severity of AKI, secondary outcomes, and limitations. Data was organized into a pre-piloted Excel (Microsoft Corp., Redmond, WA) sheet. The primary outcome was the incidence of AKI. Other outcomes included atrial fibrillation (AF), MACE, tubular biomarkers, and serious adverse events. Two reviewers extracted data independently, with a consensus process to resolve discrepancies.

Synthesis

We synthesized findings descriptively and presented them in summary tables grouped by completed studies and ongoing trials. Risk of bias was not assessed due to the scoping nature of the review.

Results

Search Results

Database searches yielded 32 unique records after removing 20 duplicates. Following title and abstract screening, 18 were excluded. Of the 14 full texts retrieved, one was unavailable, and five were excluded due to already being duplicates. Seven studies met the inclusion criteria. One study was terminated early due to achieving the desired outcome and the subsequent initiation of a larger multicentre trial. Three ongoing trials were identified from registries as seen in Figure 1.

Completed Studies

The included studies consisted of randomized controlled trials (RCTs) and observational cohort studies investigating the use of sodium-glucose cotransporter-2 inhibitors (SGLT2i) such as empagliflozin and dapagliflozin in perioperative surgical settings. Two studies were RCTs conducted in elective cardiac surgery populations, specifically patients undergoing coronary artery bypass grafting (CABG) surgery with cardiopulmonary bypass (CPB) [14,15]. Two observational studies assessed the effects of chronic SGLT2 inhibitor use in patients undergoing CABG and TAVI, respectively [16,17]. Dosing of empagliflozin ranged from 10 mg to 25 mg daily, and dapagliflozin was dosed at 10 mg daily. There was variation in SGLT2i initiation strategy; this is detailed in Table 1. The only study that continued the use of SGLT2i in the perioperative period was Snel et al. (2025) [15]. Detailed study characteristics are presented in Table 2.

**Table 1:** Sodium-glucose cotransporter-2 inhibitors initiation strategies in included studies

Author (year)	Sodium-glucose cotransporter-2 inhibitors initiation strategy
Pitta et al., 2025 [14]	Sodium-glucose cotransporter-2 inhibitors ≥3 months and stopped 72 h before coronary artery bypass graft
Makortoff et al., 2024 [16]	Sodium-glucose cotransporter-2 inhibitors initiated ≤14 d post-coronary artery bypass graft discharge
Snel et al., 2025 [15]	Sodium-glucose cotransporter-2 inhibitors 3 days prior
Paolisso et al., 2025 [17]	Sodium-glucose cotransporter-2 inhibitors ≥1 month before hospitalization

**Table 2:** Study characteristics Key: ▲ = outcome favours sodium-glucose cotransporter-2 inhibitors (p < 0.05); ▼ = favours control; ↔ = no significant difference SGLT2i: sodium-glucose co-transporter-2 inhibitor

Author (year)	Country of study	Study type	Condition and peri-op treatment tested	Sample size (n)	SGLT2i-treated patients (n)	Assessment window	Renal outcome	Selected secondary outcomes	Additional Outcomes
Makortoff et al., 2024 [16]	Canada	Observational	Type 2 diabetes mellitus post-coronary artery bypass graft discharge	1629	226	3 months and 12 months	Lower long-term acute kidney injury risk among chronic users	Major adverse cardiovascular events ↔	Safety events ↔
Pitta et al., 2025 [14]	Brazil	Randomized Controlled Trial	Type 2 diabetes mellitus who are undergoing elective coronary artery bypass graft	145	71	7 days	Reduced postoperative acute kidney injury compared with standard care ▲	Atrial fibrillation ↔ Type 5 myocardial Infarction ↔	Hospital length of stay ▲ Safety events ↔
Snel et al., 2025 [15]	Netherlands	Randomized Controlled Trial	Elective cardiopulmonary bypass cardiac surgery (mixture of coronary artery bypass graft and valves)	55	25	7 days	Reduced postoperative acute kidney injury compared with standard care ▲	Tubular biomarkers ↔ Hypoxia-inducible factor 1-alpha ▼	Serious adverse events ↔
Paolisso et al., 2025 [17]	Multinational (Europe)	Observational	Type 2 diabetes mellitus with severe aortic stenosis undergoing selective transcatheter aortic valve implantation	514; 288 with chronic kidney disease; 226 without chronic kidney disease	71 in chronic kidney disease subset; 42 without chronic kidney disease subset	48 hr + discharge	Reduced acute kidney injury events in chronic kidney disease subgroup ▲ Acute kidney injury in non chronic kidney disease subgroup ↔	Serum creatinine at hospital discharge ▲	-

Ongoing Randomized Trials

Currently, three randomized controlled trials are underway, MERCURI-2, VERTIGO, and STENOTYPE, to further investigate the impact of perioperative SGLT2 inhibitors on acute kidney injury (AKI) outcomes [18-20]. Of these, STENOTYPE is actively recruiting as of May 2025. These trials are expected to provide more definitive evidence regarding the timing, dosage, and safety of SGLT2i use in the surgical setting.

The MERCURI-2 trial aims to build upon the findings of its predecessor, MERCURI-1, which demonstrated a significant reduction in AKI incidence with perioperative empagliflozin use. By employing a multicentre, triple-blind design, MERCURI-2 seeks to validate these results across a broader patient population and surgical settings [15,18].

Similarly, the VERTIGO trial focuses on assessing the efficacy and safety of empagliflozin in preventing CSA-AKI [19]. Its design mirrors that of MERCURI-2, emphasizing the need for consistency in evaluating the potential benefits of SGLT2 inhibitors in the perioperative context.

In contrast, the STENOTYPE trial introduces a dual-primary focus: evaluating dapagliflozin not only for AKI prevention but also for reducing post-operative atrial fibrillation (POAF) in elective CABG patients [20]. While the primary endpoint is AF, AKI will be monitored as a key secondary outcome. This inclusion is highly relevant to the scope of our review, as it enables a broader understanding of the renoprotective potential of SGLT2 inhibitors even in trials not explicitly powered for AKI. It reflects the growing recognition of these agents’ multi-organ benefits in surgical patients.

Collectively, these trials will provide important insights into optimal timing, duration, and perioperative safety of SGLT2 inhibition and may help define future guidelines for their use in surgical patients. into optimal timing, duration, and perioperative safety of SGLT2 inhibition, and may help define future guidelines for their use in surgical patients. The ongoing trials are summarized in Table 3.

**Table 3:** Ongoing randomized controlled trials

Trial	Design	Country	Status	Estimated study sample	Intervention	Comparator	Patient population	Outcome
Oosterom-Eijmael et al., 2025 [18]	Phase IV, multicenter, randomized, triple-blind, placebo-controlled	The Netherlands	Active, not recruiting	784	Dapagliflozin 10 mg daily, starting 1 day pre-op to 2 days post-op	Placebo	Adults undergoing elective cardiac surgery with cardiopulmonary bypass	Incidence of cardiac surgery-associated acute kidney Injury (Kidney Disease Improving Global Outcomes criteria) within 7 days after surgery
Coca et al., 2024 [19]	Phase III, multicenter, randomized, double-blind, placebo-controlled	Spain	Ongoing	608	Empagliflozin 10 mg daily, starting 5 days pre-op to 7 days post-op	Placebo	Patients undergoing elective cardiac surgery with extracorporeal circulation	Incidence of major kidney event during the first 90 days after surgery
STENOTYPE (NCT05852704) [20]	Phase III, randomized, double-blind, placebo-controlled	Sweden, Denmark, and the Czech Republic	Recruiting	800	Dapagliflozin 10 mg daily, starting ≥7 days pre-op until hospital discharge	Placebo	Patients with chronic coronary syndrome undergoing elective coronary artery bypass graft	Incidence of postoperative atrial fibrillation and/or Acute Kidney Injury before discharge

Discussion

This scoping review highlights the potential benefits of perioperative SGLT2 inhibitors in reducing cardiac surgery-associated AKI (CSA-AKI). Consistently across included studies, SGLT2 inhibitors demonstrated a significant reduction in AKI incidence. Notably, this effect was robust across different study designs and surgical populations, underscoring the therapeutic promise of these agents.

The incidence of CSA-AKI in patients with CKD who were not treated with SGLT2 inhibitors was threefold higher than in those without CKD. Both patient groups with and without CKD demonstrated a similar risk of AKI, suggesting that SGLT2 inhibitor use alone was effective in reducing the risk of AKI associated with CKD [17].

Although the exact mechanisms behind the cardiovascular and renal advantages observed in large-scale randomized studies of SGLT2 inhibitors remain unclear, the potential mechanisms responsible for the renal protective effects of SGLT2 inhibitors (SGLT2i) proposed involve several complex pathways [8,9]. Such mechanisms are particularly relevant in cardiac surgery settings characterized by ischemia-reperfusion injury and systemic inflammatory response.

By promoting natriuresis and glucose-driven osmotic diuresis, SGLT2 inhibitors enhance sodium delivery to the macula densa, which strengthens tubuloglomerular feedback. This action triggers afferent arteriole constriction, thereby decreasing glomerular hyperfiltration, which is a process particularly susceptible to disruption during the hemodynamic shifts encountered in cardiac surgeries [21,22].

Moreover, SGLT2 inhibitors reduce oxidative stress and inflammation, as well as dampen overactivity in both the renin-angiotensin-aldosterone system and the sympathetic nervous system, all factors linked to kidney injury [22,23]. Additionally, these agents reduce peritubular fibrosis and improve tubular cell resilience against ischemic injury, thus maintaining renal structural integrity.

The reported incidence of AKI varies widely across studies due to several factors, including the type of cardiac surgery performed, differences in study populations, perioperative practices, and monitoring protocols. A contributor to this variability is the heterogeneity in AKI definitions used across the included studies. As shown in Table 4, some studies employed established criteria such as AKIN, KDIGO, or RIFLE, while others did not clearly define AKI at all. These classification systems differ in thresholds for serum creatinine change, urine output, and staging of severity, making direct comparisons of AKI incidence and severity across studies challenging. This inconsistency in outcome definitions complicates the interpretation of renal findings and underscores the need for standardized criteria in future research to allow for more meaningful synthesis and comparison of results. Definitions of AKI used by the studies are presented in Table 4.

**Table 4:** Definitions of acute kidney injury used in included studies

Author (year)	Acute kidney injury definition utilized
Makortoff et al., 2024 [16]	Not defined
Pitta et al., 2025 [14]	Acute Kidney Injury Network (AKIN) criteria; Risk, Injury, Failure, Loss of kidney function and End-stage kidney disease (RIFLE) classification; Kidney Disease Improving Global Outcome (KDIGO) classification
Snel et al., 2025 [15]	Kidney Disease Improving Global Outcome classification
Paolisso et al., 2025 [17]	Kidney Disease Improving Global Outcome classification

In the study by Pitta et al. (2025), empagliflozin was halted 72 hours before surgery (equivalent to roughly six half-lives of the drug), but there appears to be residual protective activity against kidney injury, even though significant drug activity was not anticipated during surgery [14]. Furthermore, the safety profile was reassuring, with minimal reported risks of hypoglycemia, ketoacidosis, or hypotension.

A key limitation affecting comparability across studies is the variability in the timing of SGLT2 inhibitor initiation and discontinuation. As shown in Table 1 and Table 4, some studies evaluated chronic preoperative use discontinued 72 hours prior to surgery (e.g., Pitta et al.), while others initiated therapy perioperatively (e.g., Snel et al.), or post-discharge (e.g., Makortoff et al.) [14-16]. This heterogeneity introduces significant differences in drug exposure during the perioperative window, complicating the interpretation of renoprotective effects and safety outcomes. Without standardization of timing relative to surgery, it remains difficult to isolate whether observed benefits are due to acute pharmacologic effects, longer-term conditioning, or baseline patient factors.

Despite promising results, several limitations were common across the included studies. These included small sample sizes, single-centre designs, and short follow-up periods, all of which limit the generalizability and statistical power of their findings. There was also considerable heterogeneity in the timing of SGLT2 inhibitor initiation, variability in AKI definitions (ranging from KDIGO and AKIN to undefined), and incomplete or inconsistent reporting of renal and safety outcomes. These methodological inconsistencies hinder cross-study comparison and weaken the strength of any pooled interpretations. Limitations for the studies are included in Table 5.

**Table 5:** Summary of methodological limitations in included studies eGFR: estimated glomerular filtration rate; HbA1c: glycated hemoglobin

Author (year)	Methodological/design limits	Population/external-validity limits	Intervention limits
Makortoff et al., 2024 [16]	• Retrospective registry, no randomization; residual confounding despite multivariable and Inverse Probability of Treatment Weighting adjustment. • Possible immortal-time bias (sodium-glucose cotransporter-2 inhibitors dispensing within 14 d).	• Canadian provincial cohort; 86% male, mean age ≈65 → sex and ethnicity under-represented. • Sodium-glucose cotransporter-2 inhibitors users healthier at baseline (lower HbA1c, better left ventricular ejection fraction).	• Any sodium-glucose cotransporter-2 inhibitors agent, dose and adherence not standardised; exposure started post-discharge → not perioperative. • Adherence bias: only 77% of users achieved a ≥80% proportion of days covered; analysis relied solely on pharmacy dispensing data.
Pitta et al., 2025 [14]	• Single-centre, pragmatic, open-label randomized controlled trial (blinded adjudication only).	• All participants had type 2 diabetes mellitus and elective on-pump coronary artery bypass graft → limits generalisability to non-diabetics, urgent cases, valves/off-pump surgery.	• Empagliflozin stopped 72 h pre-op (per FDA guidance) → true perioperative exposure uncertain. • Fixed 25 mg dose; no titration to eGFR.
Snel et al., 2025 [15]	• Small (N = 55), single-centre, open-label pilot; no allocation concealment. • Imbalance in baseline eGFR despite randomization.	• Elective cardiopulmonary bypass surgery only; 73% male; exclusion of eGFR <30 mL/min → not applicable to advanced chronic kidney disease.	• Empagliflozin 10 mg OD for 3 days pre-op → 2 days postop; regimen may be sub-therapeutic for renal endpoints. • No comparator active-control.
Paolisso et al., 2025 [17]	• Prospective observational; propensity methods reported but unmeasured confounding likely. • Multinational but data entry heterogeneous.	• Study limited to type 2 diabetes mellitus and severe aortic stenosis • Exclusion of eGFR <30 mL/min → not applicable to advanced chronic kidney disease.	• Mixed sodium-glucose cotransporter-2 inhibitors; timing and duration not captured. • Concurrent nephrotoxic management not standardized.

Future research, notably the ongoing large-scale, multicenter, randomized controlled trials, MERCURI-2, VERTIGO, and STENOTYPE, will be critical in establishing definitive recommendations [18-20]. Collectively, these trials underscore a growing recognition of the potential reno- and cardioprotective effects of SGLT2 inhibitors in the surgical setting. Their outcomes could significantly influence perioperative management protocols, offering a pharmacological approach to mitigate the risks of CSA-AKI.

In addition to the limitations of the included studies, this scoping review has several inherent constraints. Most notably, it involved no formal critical appraisal of study quality, consistent with scoping review methodology, which limits the ability to assess internal validity. There is also the potential for publication bias, as ongoing or unpublished studies with neutral or negative findings may not have been captured.

## Conclusions

This scoping review highlights a growing body of early evidence suggesting that SGLT2 inhibitors may offer renoprotective benefits in the context of cardiac surgery. Completed studies, though limited in size and methodological consistency, consistently report a reduction in CSA-AKI incidence without clear safety concerns. However, significant limitations remain, including heterogeneity in AKI definitions and timing of drug administration. Ongoing large-scale randomized trials are expected to provide the definitive evidence needed to guide safe and effective perioperative use of SGLT2 inhibitors. Until then, their routine initiation solely for CSA-AKI prevention remains investigational.

## Figures and Tables

**Figure 2:**
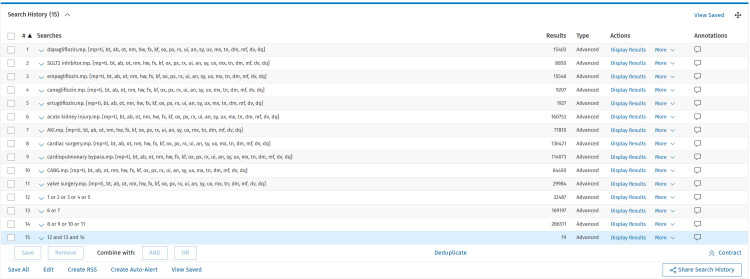
OVID search

**Figure 3:**
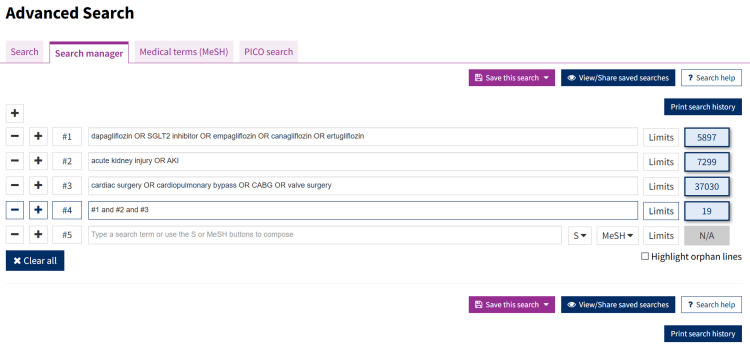
Cochrane Library advanced search

**Figure 4:**
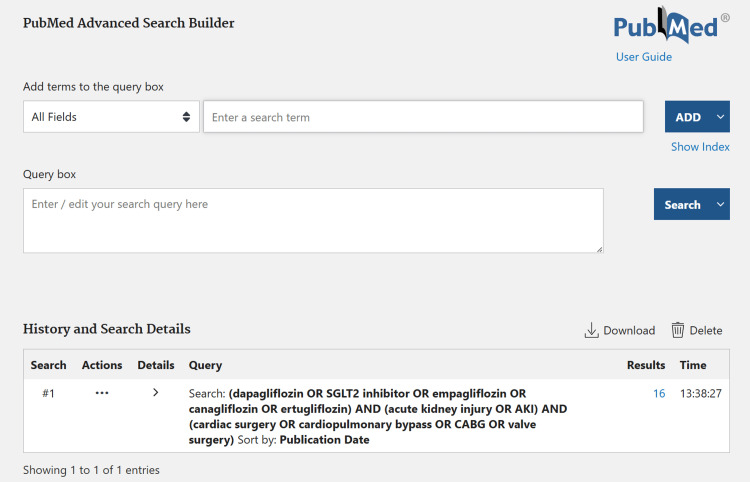
Boolean for PubMed

## Appendices

Appendix 1

Appendix 2 

Appendix 3 
